# *BCL2* promoter region mutations are an independent marker of *BCL2* level in lymphoid malignancies

**DOI:** 10.1038/s41525-026-00590-z

**Published:** 2026-06-19

**Authors:** Daniel Kuznicki, Paulina Galka-Marciniak, Malwina Suszynska, Piotr Kozlowski

**Affiliations:** Institute of Bioorganic Chemistry, Polish Academy of Sciences, Poznan, Poland

**Keywords:** Cancer, Oncology

## Abstract

*BCL2* upregulation is a key driver of lymphomagenesis and treatment response. By analyzing large-scale whole-genome datasets, we identify a lymphoma-specific noncoding mutational hotspot encompassing the *BCL2* promoter that, independent of other factors such as the t(14;18) translocation, strongly associates with allele-specific *BCL2* upregulation. These mutations disrupt regulatory protein-binding sites, alter *BCL2* isoform balance, and associate with worse survival in diffuse large B-cell lymphoma.

Upregulation of *BCL2* is a key factor driving lymphoma and affecting its therapy. Prevailing models attribute *BCL2* overexpression to the t(14;18)(q32;q21) (t(14;18)) translocation in B-cell non-Hodgkin lymphomas (BNHL) and to the loss of miR-15a/16-1 in chronic lymphocytic leukemia (CLL)^1^. However, the contribution of somatic mutations within the *BCL2* promoter regions remains underexplored. This oversight is largely due to the challenges of analyzing mutations in noncoding regions and the confounding effects of somatic hypermutation, which results in omitting the region in whole-genome analyses^2^ or focusing on coding non-synonymous mutations, thereby neglecting analysis of most mutations in the region^3,4^.

## Recurrent mutation hotspot in the *BCL2* promoter region in B-cell lymphomas

**Fig. 1:**
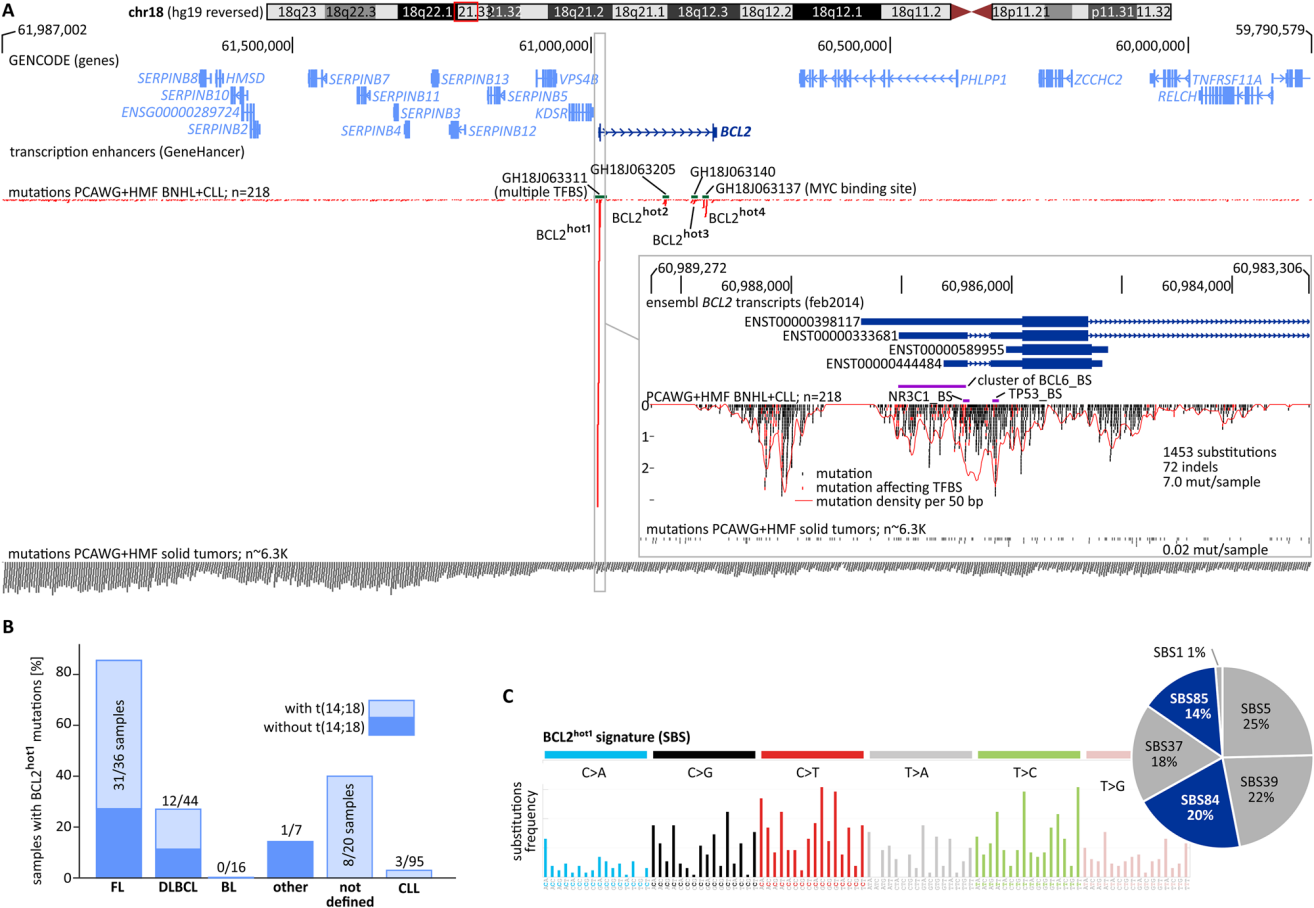
Landscape of *BCL2* mutations in lymphoid malignancies and solid tumors. **A** Genomic localization of *BCL2* on chromosome 18, illustrating the distribution of somatic mutations in lymphoid malignancies (PCAWG + HMF BNHL + CLL) and solid tumors (the graph was prepared using UCSC Genome Browser with the ‘squish’ mode for mutation visualization). GeneHancer enhancer elements overlapping with mutational hotspots (BCL2^hot1–4^) are indicated as green bars. The inset shows a zoomed-in view of the BCL2^hot1^ region with indicated mutation density (in a 50 bp smoothing window) and mutations predicted by FunSeq2^6^ to disrupt TFBSs (marked in red). BCL6, TP53, and NR3C1 repressor binding sites (BS) are indicated as purple bars. **B** Frequency of lymphoid malignancy samples with BCL2^hot1^ mutations, stratified by subtype. The proportion of samples with and without t(14;18) is shown in light and dark blue, respectively. **C** Mutational signature graph showing proportion of all substitution types (in +/-1 nucleotide context) in BCL2^hot1^. The pie chart displays the proportion of substitutions attributed to established mutational signatures; signature SBS84 and SBS85, associated with AID activity, are highlighted in dark blue. Signature analysis was performed using SigProfilerAssignment.

Through analysis of noncoding regions in whole-genome sequencing data from ~2000 Pan-Cancer Analysis of Whole Genomes (PCAWG) cancer samples, we identified a high density of somatic mutations upstream of the *BCL2* gene. Further analysis showed that the hotspot (hereafter called BCL2^hot1^) spans ~6 kb from the promoter through exon 1 (including the 5’ untranslated region (UTR) and the sequence encoding the first 45 amino acids) to the first ~2 kb of intron 1 (chr18:60,983,306-60,989,272 (GRCh37/hg19; Fig. 1A). This region coincides with GeneHancer transcription factor binding sites (TFBS) rich region and a previously identified *BCL2* superenhancer^5^ (Fig. 1A).

BCL2^hot1^ mutations were present in 44% of BNHL (*n* = 98) and ~3% of CLL (*n* = 95) samples, with 1-75 mutations per sample. We confirmed a similar frequency/distribution of BCL2^hot1^ mutations in a small cohort (*n* = 25) of metastatic BNHL samples from Hartwig Medical Foundation (HMF; accession: DR-275). Among the BNHL subtypes, mutations were the most frequent in follicular lymphoma (FL; 83%), less frequent in diffuse large B-cell lymphoma (DLBCL; 27%), and absent in Burkitt lymphoma (BL) (Fig. 1B), which may reflect their biology related to exposure to activation-induced cytidine deaminase (AID) mutagenic activity in germinal centers (GC). Whereas FL is a prototypical GC-derived, indolent, slow-growing malignancy characterized by prolonged GC residence and therefore the highest AID exposure, DLBCL is a more diverse and aggressive type, with varying exposure to the GC microenvironment. Although BL also originates from GC B cells, it is mainly driven by MYC activation (translocation), leading to rapid proliferation and reduced exposure to AID. The mutations were virtually absent in thousands of solid tumors (except for one HMF sample (CPCT02070117T) with 30 mutations, designated as prostate cancer metastasized to a lymph node) and did not occur in acute myeloid leukemias (AML; *n* = 16) and myeloproliferative neoplasms (MPN; *n* = 27).

The analysis of selection pressure (mutation enrichment) in PCAWG classified the BCL2^hot1^ region (mutations) as a highly-potent cancer driver: ActiveDriverWGS, which models the local background mutation rate while accounting for genomic covariates, including TFBS, identified the BCL2^hot1^ region as a highly significant driver hotspot (*q* = 3.01e-31). Subsequently, the functional enrichment and driver status of BCL2^hot1^ mutations were demonstrated using OncodriveFML, with CADD and FATHMM-XF scores (*q* = 0.016 and 0.002, respectively), and Mutsig2CV_NC^2^ *q* = 0.008. Similar results, although less significant due to a smaller sample size, were observed in HMF samples.

## BCL2^hot1^ mutations disrupt transcription factor binding sites and exhibit non-random mutational signatures

Analyses of BCL2^hot1^ mutations, using the FunSeq2^6^ framework, based on ENCODE ChIP-seq data, predicted that multiple of the mutations disrupt key TFBSs (Fig. 1A), including CTCF, E2F1, EBF1, EGR1, ELF1, FOXA1, FOXA2, NFKB1, TAF1, and ZEB1. The GeneHancer database annotates the BCL2^hot1^ region as GH18J063311, containing over 150 TFBS. Additionally, it was experimentally demonstrated that aberrations in the binding sites of repression factors BCL6^7^, TP53^8^, and NR3C1^5^ in the *BCL2* 5’UTR negatively affect *BCL2* repression. Since these abnormalities match mutations identified in this study (Fig. 1A), they may also account for the effects observed here. For instance, in 7 DLBCL samples, we found mutations that were reported as *NR3C1* variants affecting *BCL2* expression in DLBCL samples without t(14;18)^5^. Additionally, many of these factors are actively transcribed, and some are differentially expressed in BNHL samples with and without BCL2^hot1^ mutations (Fig. S1).

An interesting observation in this context, though independent of the promoter mutations, is the presence of three smaller intragenic mutation hotspots located in the *BCL2* intron (BCL2^hot2-hot4^), coinciding with GeneHancer high-confidence enhancers, one of which includes a MYC binding site (Fig. 1A).

Mutational signature analysis showed that only 20% and 14% of BCL2^hot1^ variants are attributed to direct and indirect effects of AID (single base substitutions (SBS) signature SBS84 and SBS85, respectively; Fig. 1C). The signature pattern was generally consistent across different cancer types (DLBCL vs. FL vs. all; Fig. 1C and Fig. S2), suggesting a common mutagenic mechanism among lymphoma subtypes. The comparison of mutation signatures assigned to mutations identified as affecting or not affecting TFBS showed that TFBS-affecting mutations tend to be depleted of SBS84 (OR = 0.72, Fisher test *p* = 0.039), but are enriched in SBS85 (OR = 1.41, *p* = 0.058). On the other hand, recent studies suggest that AID-mediated hypermutagenesis is not random but preferentially targets active superenhancers^5,9^ and TFBSs^10^, underscoring the significance of the targeted regions. It is important to note, however, that the concept of mutation signatures was originally developed to analyze mutational profiles within individual samples and may not accurately reflect mutations in a specific hotspot characterized by non-random nucleotide composition and/or undergoing selective pressure. Consequently, assigning canonical signatures to individual mutations is prone to minor fluctuations in substitution-type ratios, especially occurring in small mutation sets like those examined in this study.

## BCL2^hot1^ mutations associate with allele-specific *BCL2* overexpression and DLBCL poor prognosis

**Fig. 2:**
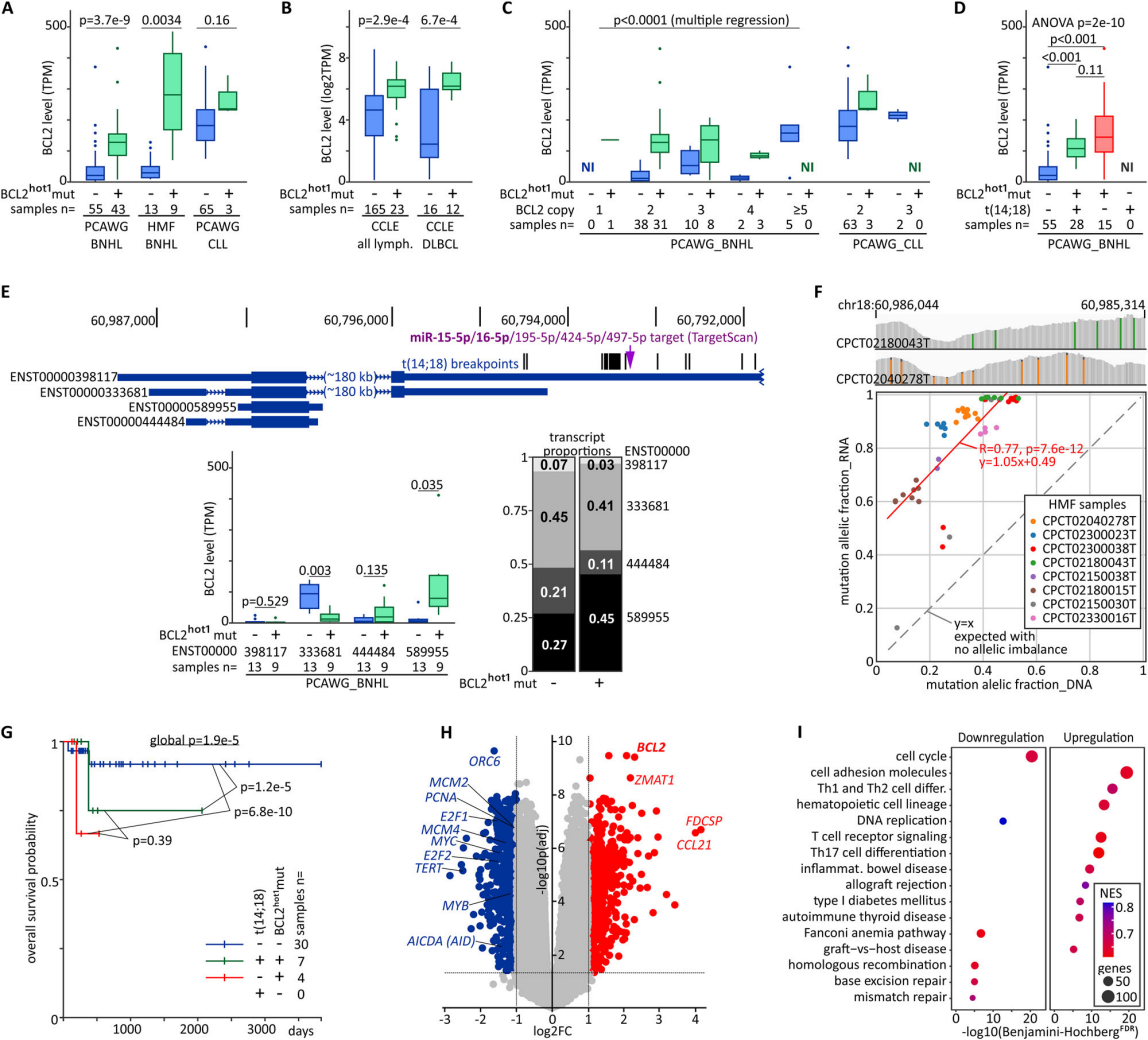
Associations of BCL2^hot1^ mutations in lymphoid malignancies. **A** *BCL2* levels in PCAWG BNHL, HMF BNHL, and PCAWG CLL cohorts with (+) and without (−) BCL2^hot1^ mutations. Sample sizes are indicated below the charts (also in **B**–**D**, **E**, **G**). **B** *BCL2* levels (log_2_ Transcripts Per Million (TPM)) in Broad Institute CCLE lymphoid and DLBCL cell lines with and without BCL2^hot1^ mutations. Note that, as CCLE utilizes whole-exome sequencing, the identified mutations primarily localize to the *BCL2* first exon. **C** *BCL2* levels in PCAWG BNHL, and PCAWG CLL samples stratified by the presence of BCL2^hot1^ mutations and *BCL2* copy numbers. NI (not identified) indicates groups where no samples were found (also in **D**). **D** *BCL2* levels in BNHL samples stratified by the presence of BCL2^hot1^ mutations and t(14;18). The overall ANOVA and adjusted post-hoc p-values are indicated. **E** The impact of BCL2^hot1^ mutations on *BCL2* isoform levels. (Above) Scheme of *BCL2* isoforms showing t(14;18) breakpoints (black bars) and miR-15a/16-1 binding sites (purple arrowhead). (Below) BCL2 isoform levels in BNHL samples with and without BCL2^hot1^ mutations, with the nominal (unadjusted for multiple comparisons) p-values. (Right) Stacked-bar chart of isoform proportions in samples with and without BCL2^hot1^ mutations. **F** *BCL2* allelic imbalance in 8 DLBCL HMF samples with BCL2^hot1^ mutations. (Above) RNA-seq coverage of the *BCL2* constitutive exonic sequence within BCL2^hot1^ in two representative samples. Light-gray bars indicate nucleotide coverage; colored bars indicate coverage of mutant alleles; dark-gray bars indicate coverage of wild-type and unannotated variant alleles. (Below) Correlation of mutant allelic frequencies at DNA (x-axis) and RNA (y-axis) levels. Colors indicate mutations identified in different samples. **G** Kaplan–Meier survival analysis of DLBCL patients across mutually exclusive (at sample level) cohorts: without BCL2^hot1^ mutations and t(14;18) (dark blue line), with BCL2^hot1^ mutations and t(14;18) (green line), and with BCL2^hot1^ mutations only (red line). Significance was assessed using the log-rank test; vertical ticks indicate censoring times. **H** Volcano plot of differentially expressed genes in PCAWG BNHL samples with vs. without BCL2^hot1^ mutations. **I** GSEA of differentially expressed genes shown in **H** (NES Normalized Enrichment Score, FDR False Discovery Rate).

The comparison of samples with and without mutations revealed that the presence of mutations is strongly associated with *BCL2* expression, with a median fold change (FC) = 5 and FC = 1.2, in PCAWG BNHL and CLL samples, respectively, and FC = 9.5 in HMF BNHL samples (Fig. 2A). Interestingly, basal *BCL2* levels were approximately 10 times higher in CLL than BNHL, potentially explaining the smaller effect of the mutations in CLL. To further validate our findings, we analyzed 1955 cell lines from the Broad Institute Cancer Cell Line Encyclopedia (CCLE). The analysis identified 83 BCL2^hot1^ mutations, mostly in lymphoid cell lines (29/36, 80.5%), including DLBCL cell lines (15/29, 51.7%), and rarely in other cell lines (7/1955, 0.4%). Consistent with the previous analyses, lymphoid cell lines with BCL2^hot1^ mutations exhibited significantly higher *BCL2* expression than wild-type lines (FC = 1.3, p = 2.9e-4). The effect was even more pronounced in DLBCL samples analyzed separately (FC = 2.5, p = 6.7e-4; Fig. 2B).

Subsequent analyses showed that the effect of BCL2^hot1^ mutations (*r*_partial_ = 0.6, *p* < 0.0001) on *BCL2* level is independent of and stronger than the effects of *BCL2* copy number (*r*_partial_ = 0.3, *p* = 0.0026; multiple regression *p* < 0.0001) (Fig. 2C) and t(14;18) (Fig. 2D). Notably, 15 PCAWG BNHL samples with BCL2^hot1^ mutations exhibited even higher *BCL2* levels than samples with mutations and the translocation, which is consistent with^11^, where the authors reported *BCL2* overexpression in the absence of t(14;18) in FL samples. These findings challenge the suggested “enhancer hijacking” effect, which attributes *BCL2* overexpression to its juxtaposition with the Eµ enhancer of the *immunoglobulin heavy chain 6* (*IGHJ6*) gene. Although the effect of the translocation on the expression of surrounding genes cannot be completely ruled out, analysis of *IGHJ6* (on chromosome 14) and genes flanking *BCL2* also does not show a distinctive effect of the translocation (Fig. S3). On the other hand, the translocation may disrupt miR-15/16 binding sites in the *BCL2* 3′UTR^1^, relieving *BCL2* repression and potentially explaining the clustering of breakpoints upstream of miR-15/16 binding sites (Fig. 2E[above]). We also found that the increase of *BCL2* expression is independent of the number of mutations in BCL2^hot1^ (Fig. S4).

The comparison of genomic and transcriptomic allelic fractions of BCL2^hot1^ mutations located in the constitutive exonic sequence (chr18:60,985,314-60,986,045) in 8 HMF DLBCL samples revealed a very strong allelic imbalance in favor of the mutated alleles (Fig. 2F). The majority of mutations showed very strong allelic enrichment, representing >90% of transcript reads, with a median enrichment of mutated alleles at the RNA vs. DNA level across all mutations of 11 (ranging from 2.5 to 88 in individual samples). Moreover, whole-genome allelic-specific expression analysis using phASER^12^ identified *BCL2* with very high confidence (−log_10_p(adjusted) from 54 to 320 across individual samples) as the top-ranked gene exhibiting allelic imbalance. The allelic imbalance analysis demonstrated that the effect of BCL2^hot1^ mutations is allele-specific.

Further analysis of transcript levels in the HMF dataset suggests that BCL2^hot1^ mutations may have an unequal effect on different *BCL2* splicing isoforms (Fig. 2E[below]). The observed trend indicates an increase in ENST00000589955 (FC = 11.8), the isoform missing the 2nd exon encoding the C-terminal domain responsible for interaction with the mitochondrial membrane and activation of apoptosis, but retaining the N-terminal domain taking part in angiogenesis^13^, at the expense of all other isoforms.

The analysis of the effect of mutation occurrence on the overall survival of DLBCL patients revealed that BCL2^hot1^ mutations are associated with reduced survival, independent of t(14;18) (Fig. 2G), consistent with findings from the GOYA study, which demonstrated a similar effect for *BCL2* coding mutations^14^. Due to an insufficient number of samples with available clinical data in specific subgroups, such as the lack of control samples without BCL2^hot1^ mutations in FL, survival analysis could not be performed for other lymphoma subtypes.

The differential transcriptomic analysis of PCAWG BNHL samples with BCL2^hot1^ mutations revealed 652 upregulated (including *BCL2*) and 549 downregulated genes (Fig. 2H). This analysis identified a robust transcriptomic shift toward cell cycle suppression and reduced DNA replication. Key drivers of cell cycle progression were among the most deeply downregulated genes, including *E2F2*, *MYC*, *CDC25A*, and *E2F1*. The suppression of *E2F1* is particularly interesting, as it binds the *BCL2* 5′UTR^15,16^, suggesting a feedback loop regulation of *BCL2* expression. Furthermore, essential DNA replication machinery was significantly suppressed, including components of the Minichromosome Maintenance complex (*MCM4* and *MCM2*), the Origin Recognition Complex (*ORC6*), and the standard replication marker *PCNA*. Conversely, we observed a slight upregulation of well-established cell cycle inhibitors and classical markers of G_0_/G_1_ arrest, such as *CDKN1B* and *RBL2*. In addition to these proliferation dynamics, other notable cancer-related genes, such as *TERT* and AID encoding *AICDA*, were significantly downregulated (Fig. 2H). Gene Set Enrichment Analysis (GSEA) revealed pronounced downregulation of cell cycle and DNA replication pathways, and upregulation of cell adhesion and hematopoietic differentiation in BCL2^hot1^ samples (Fig. 2I).

## Hypothetical mechanisms and next steps to elucidate the role of BCL2^hot1^ mutations in lymphomagenesis

A simultaneous loss of DNA repair and division capacity may reflect a condition where TP53, in response to DNA damage, arrests cell proliferation but loses its ability to induce apoptosis, instead directing and maintaining the cell in a state of senescence. Such a condition, for example, was observed in a mouse model with the TP53 R172P mutation and was linked to activation of the antiproliferative p21 (*CDKN1A*) pathway^17^, and may be linked with aneuploid and senescent cells, a mitotic escape mechanism in DLBCL that contributes to drug resistance^18^. We also hypothesize that the observed transcriptomic patterns reflect the CLL phenotype, in which elevated BCL2 levels confer resistance to apoptosis but limit proliferation and motility. It was reported that *BCL2* overexpression stabilizes Bcl2–gelsolin complexes, enhancing actin polymerization and inhibiting cell motility and spreading^19^, while delaying the G_0_/G_1_–S transition and suppressing proliferation^20^. As a result, the BCL2 inhibitor venetoclax, when used to treat CLL, effectively induces apoptosis but may also trigger tumor lysis syndrome due to abruptly disrupting BCL2-mediated suppression of cell motility. Additionally, the *BCL2* isoform analysis suggests that the mutations may influence promoter preference, particularly promoting the proangiogenic^13^ and antiapoptotic properties of BCL2.

In summary, we identified and characterized the genomic context of BCL2^hot1^, a *BCL2* promoter-region mutational hotspot that is frequently mutated in lymphomas, particularly in FL and DLBCL. We also provided strong statistical evidence for an association between BCL2^hot1^ mutations and increased *BCL2* expression, largely independent of other factors. However, explaining the mechanism of this association or demonstrating the causal role of BCL2^hot1^ mutations in increasing *BCL2* expression would require extensive functional studies, including testing independent mutations, e.g., generated with CRISPR-based approaches, in various cellular and animal models, and linking BCL2^hot1^ mutations with variants present in the transcript using long-read sequencing or other genetic approaches to track transcripts from specific alleles. Specifically, assays measuring cell cycle kinetics, actin polymerization via flow cytometry, and real-time migration will help confirm whether these non-coding mutations directly drive the observed shift in cellular behavior. Additionally, to better understand the role of BCL2^hot1^ mutations in regulating *BCL2* levels, the relationship between BCL2^hot1^ mutations and t(14;18), as well as the potential of BCL2^hot1^ mutations as biomarkers for clinical outcomes—such as patient survival, drug sensitivity/resistance, or toxicity—requires analyzing of more, well-characterized lymphoma samples, especially to identify and study additional samples with BCL2^hot1^ mutations but lacking t(14;18).

## Supplementary information

Figure_S1_S4

## Data Availability

All data analyzed in this study are publicly available. Whole-genome sequencing and RNA-sequencing data, together with associated clinical metadata, from the PCAWG project are available through the ICGC Data Portal (https://platform.icgc-argo.org/). Whole-genome sequencing, RNA-sequencing, and clinical data from the Hartwig Medical Foundation are available via the Hartwig Medical Foundation data portal (https://www.hartwigmedicalfoundation.nl) upon approval of a data access request. Preprocessed RNA-sequencing data were additionally obtained from the UCSC Xena platform (https://xenabrowser.net) and the EMBL-EBI Expression Atlas (https://www.ebi.ac.uk/gxa). Somatic variants and transcriptomic data of cell lines were obtained from the Broad Institute Cancer Cell Line Encyclopedia (CCLE) via the depmap.org portal. No new data were generated in this study.
