# Supplementary material for: BCL2 promoter region mutations are an independent marker of BCL2 level in lymphoid malignancies
Source: NPJ Genom Med. 2026 Jun 19;11:35. doi: 10.1038/s41525-026-00590-z (PMC13279811; doi:10.1038/s41525-026-00590-z)
Supplement: Supplementary file 1 — Figure_S1_S4 [file 41525_2026_590_MOESM1_ESM.pdf]

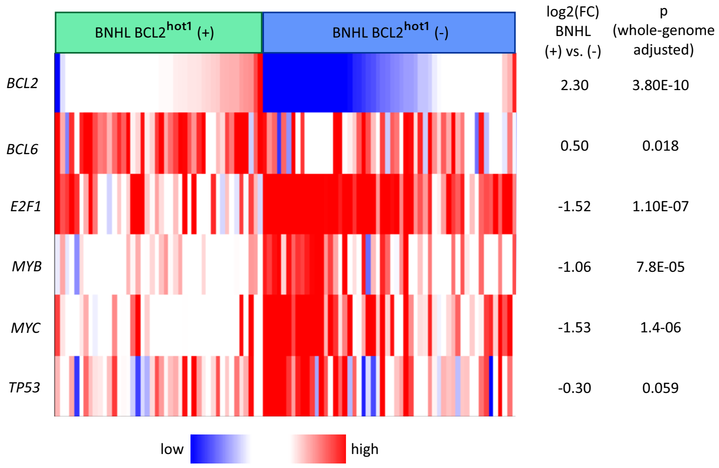

Figure S1. Heatmap showing expression levels of *BCL2* and a panel of relevant regulatory genes (indicated on the left) in PCAWG BNHL samples with and without BCL2^{hot1} mutations. The samples are sorted according to the *BCL2* level. Log_2FC and the corresponding p values (adjusted for whole-genome differential expression analysis) of differential levels in BNHL samples with and without BCL2^{hot1} mutations are indicated on the right.

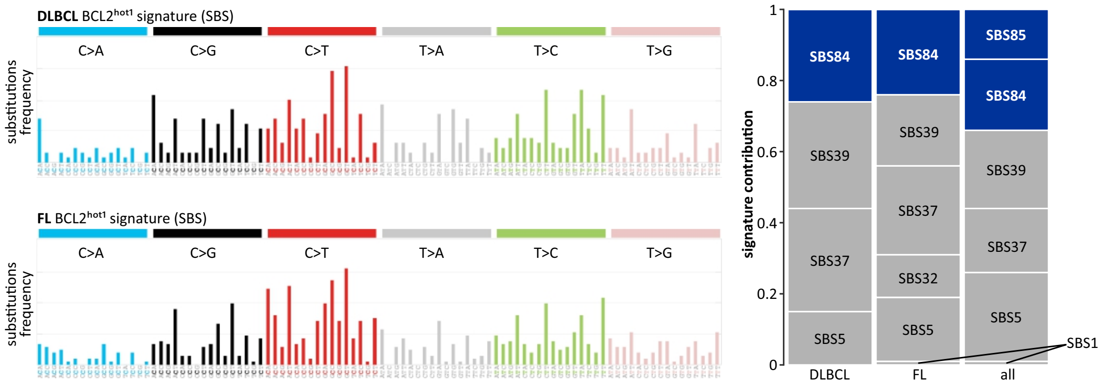

Figure S2. Mutational signature graphs showing proportion of all substitution types (in +/-1 nucleotide context) in BCL2^{hot1}, separately in DLBCL and FL. (Right) The stacked-bar chart showing the proportion of substitutions attributed to established mutational signatures in DLBCL, FL, and all lymphoma samples (as in Fig. 1C); signature SBS84 and SBS85, associated with AID activity, are highlighted in dark blue.

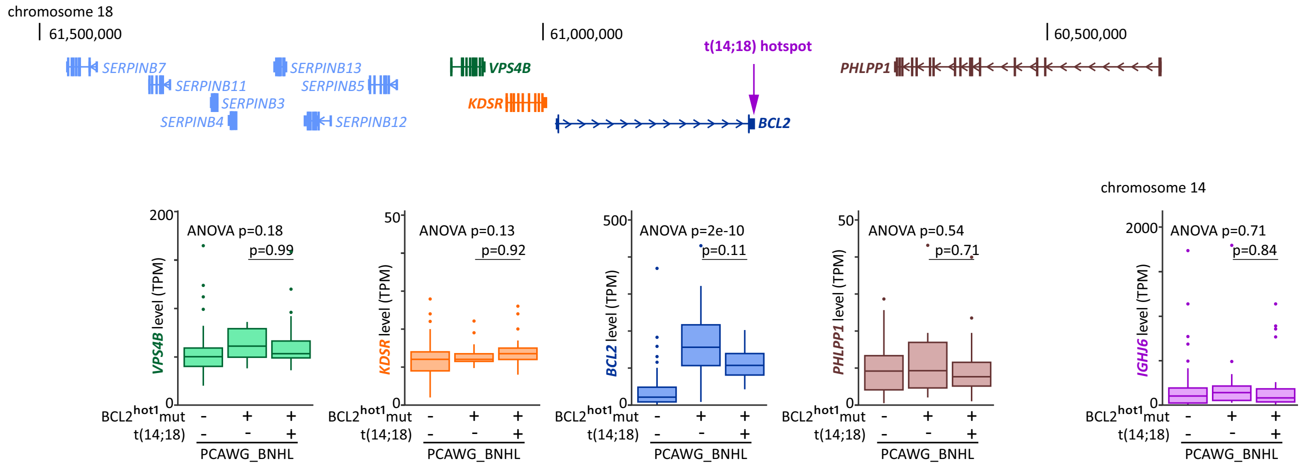

Figure S3. The expression levels (in TPM) of genes located in proximity to the t(14;18) hotspot (purple arrowhead) in PCAWG BNHL samples, stratified by the occurrence of BCL2^{hot1} mutations and t(14;18): the *IGHJ6* immunoglobulin gene located at chromosome 14 (not shown on the map above), two genes located at chromosome 18 upstream of *BCL2* (*KDSR* and *VPS4B*), and *PHLPP1* located downstream of *BCL2*. The serpin genes (shown on the left in blue) are not expressed in BNHL samples. The overall p-values for ANOVA and the post-hoc test p-values (adjusted for multiple testing) for samples with BCL2^{hot1} mutation and with both t(14;18) and BCL2^{hot1} mutations are shown on the graphs. P-values were not adjusted for comparisons between genes.

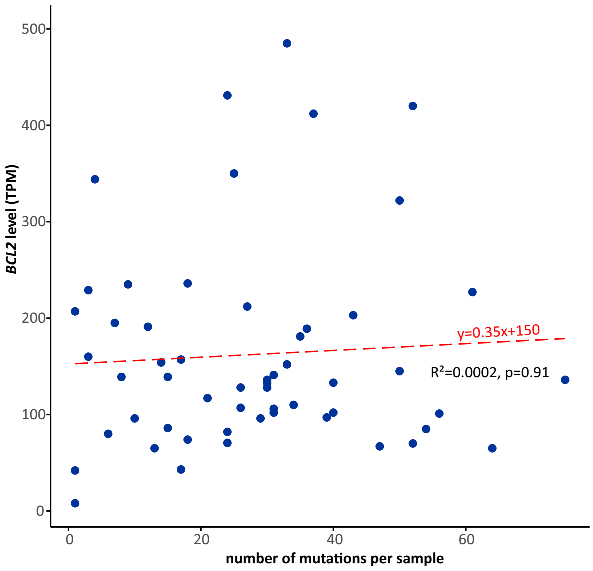

Figure S4. Correlation between the number of BCL2^{hot1} mutations and the *BCL2* level in BNHL samples with BCL2^{hot1} mutations; each dot represents an individual sample; a red dashed line denotes the linear trend.
